# Strontium-doped chromium oxide for RhB reduction and antibacterial activity with evidence of molecular docking analysis

**DOI:** 10.3389/fchem.2023.1167701

**Published:** 2023-04-13

**Authors:** Muhammad Ikram, Anum Shahzadi, Muhammad Bilal, Ali Haider, Anwar Ul-Hamid, Walid Nabgan, Junaid Haider, Salamat Ali, Francisco Medina, Muhammad Imran

**Affiliations:** ^1^ Solar Cell Applications Research Lab, Department of Physics, Government College University Lahore, Lahore, Pakistan; ^2^ Faculty of Pharmacy, The University of Lahore, Lahore, Pakistan; ^3^ Department of Clinical Sciences, Faculty of Veterinary and Animal Sciences, Muhammad Nawaz Shareef University of Agriculture, Multan, Pakistan; ^4^ Core Research Facilities, King Fahd University of Petroleum and Minerals, Dhahran, Saudi Arabia; ^5^ Departament d’Enginyeria Química, Universitat Rovira i Virgili, Tarragona, Spain; ^6^ Chinese Academy of Sciences, Tianjin Institute of Industrial Biotechnology, Tianjin, China; ^7^ Department of Physics, The University of Lahore, Lahore, Pakistan; ^8^ Government College University Faisalabad, Sahiwal, Punjab, Pakistan

**Keywords:** dye degradation, MDR *E. coli*, RhB, antibacterial, Cr_2_O_3_

## Abstract

The emergence of multi-drug resistance (MDR) in aquatic pathogens and the presence of cationic dyes are the leading causes of water contamination on a global scale. In this context, nanotechnology holds immense promise for utilizing various nanomaterials with catalytic and antibacterial properties. This study aimed to evaluate the catalytic and bactericidal potential of undoped and Sr-doped Cr_2_O_3_ nanostructures (NSs) synthesized through the co-precipitation method. In addition, the morphological, optical, and structural properties of the resultant NSs were also examined. The optical bandgap energy of Cr_2_O_3_ has been substantially reduced by Sr doping, as confirmed through extracted values from absorption spectra recorded by UV-Vis studies. The field-emission scanning electron microscopy (FE-SEM) and transmission electron microscopy (TEM) micrographs illustrate that the composition of Cr_2_O_3_ primarily consisted of agglomerated, irregularly shaped NSs with a morphology resembling nanoflakes. Moreover, the presence of Sr in the lattice of Cr_2_O_3_ increased the roughness of the resulting NSs. The catalytic activity of synthesized NSs was analyzed by their reduction ability of Rhodamine B (RhB) dye in the dark under different pH conditions. Their antibacterial activity was evaluated against MDR *Escherichia coli* (*E. coli*). Sr doping increased antibacterial efficiency against MDR *E. coli*, as indicated by inhibition zone measurements of 10.15 and 11.75 mm at low and high doses, respectively. Furthermore, a molecular docking analysis was conducted to determine the binding interaction pattern between NSs and active sites in the target cell protein. The findings corroborated antimicrobial test results indicating that Sr-Cr_2_O_3_ is the most effective inhibitor of FabH and DHFR enzymes.

## 1 Introduction

The development of multi-drug resistance (MDR) in pathogenic bacteria has evolved significantly in recent years (Franci et al., 2015). A team from the University of Alberta has discovered that a strain of *E. coli* (*Escherichia coli*) bacterium can survive and flourish in wastewater treatment plants. Although oxygenation, chlorine, and other treatments in sewage facilities are effective in eradicating the majority of *E. coli*, researchers have identified certain pathogenic strains of *E. coli* that exhibit significant resistance to these treatments. The efficacy of conventional antibiotics diminishes over time as bacteria resist them (Wise and BSAC Working Party on The Urgent Need: Regenerating Antibacterial Drug Discovery and Development, 2011). This poses a significant threat to the health and lives of millions of people each year. In addition, the progress of industrialization and urbanization generates a significant volume of waste in the form of dyes, heavy metals, and microorganisms continuously discharged into rivers and soil (Chen et al., 2015; Sallam et al., 2018). Every year, numerous types of dyes released from different sectors worldwide are dumped into aquatic systems (He et al., 2019). These are colored chemicals, and their pollution is primarily attributed to their high stability, which stems from the presence of numerous aromatic rings. Rhodamine B (RhB) is an amino xanthene dye that finds widespread applications in the colored glass industry, as well as in the fields of textiles, biology, and fluorescent staining. RhB is commonly detected in dye effluent as it is widely used in the textile industry (Qamar et al., 2020; Nguyen et al., 2021). Textile dyes have been shown to increase toxicity, reduce photosynthesis, impair plant growth, enter the food chain, and promote carcinogenic effects in water (Magureanu et al., 2008; Hameed and Ismail, 2019; He et al., 2019; Eltaweil et al., 2022). Consequently, the simultaneous eradication of organic contaminants prevalent in industrial wastewater, including chemicals, phenolic compounds, colorful dyes, and microorganisms, could effectively replace typical phased treatment methods.

Recently, metal and metal oxide nanoparticles (NPs) gained significant medical and health applications due to their superior stability at higher temperatures and pressures than conventional organic antimicrobials (Liu et al., 2009; Di et al., 2017). Transition metal NPs have been the subject of extensive research due to their potent antibacterial properties. Consequently, nanostructured chromium oxide (Cr_2_O_3_) with large surface areas attracted researchers worldwide (El-Sheikh et al., 2009). In contrast to traditional polycrystalline materials, they display advantageous and unique features. Shafi et al. reported Cr_2_O_3_ NPs with Brunauer–Emmett–Teller (BET) area of 219.9 m^2^g^−1^ and pore width of 4.2 nm (Shafi et al., 2021). The majority of research conducted on the antibacterial properties of Cr_2_O_3_ NPs has focused on their effectiveness against Gram-negative bacteria, using *E. coli* as a representative model (Ramesh et al., 2012; Almontasser et al., 2021; Ghotekar et al., 2021). Chromium and supported chromium oxides have been utilized in various catalytic processes such as the dehydrogenation of toluene, the decomposition of ammonia, and the oxidation of toluene. Calcined chromia catalysts, both supported and unsupported, exhibit excellent activity in redox processes (El-Sheikh et al., 2009). Additionally, supported chromium oxide catalysts have found applications in selective catalytic reduction of NOx with ammonia, polymerization reactions, and oxidative dehydrogenation of isobutene (Abu-Zied, 2000). Studies on chromium oxide catalysts have shown that the type and concentration of surface Cr-O species play a significant role in controlling their adsorptive and catalytic characteristics (Fouad et al., 1991; Gabr et al., 1994). Doping Cr_2_O_3_ with metals has enhanced its catalytic and antibacterial properties. Rare Earth metals can readily form strong bonds with functional groups when used as dopants in metal oxides, owing to the availability of 4f empty orbitals (Mehtab et al., 2022). Strontium (Sr) is a chemically highly reactive alkaline Earth metal that exhibits chemical similarity with calcium and can be utilized to enhance the properties of Cr_2_O_3_. The utilization of Sr in various applications has been extensively investigated, revealing its potential for beneficial effects through doping with metal oxides (Akihide et al., 2004; Li et al., 2007; Ramam and Chandramouli, 2009; Suresh and Roy, 2012; Kiani et al., 2020).

Numerous methods, including solid thermal decomposition (Li et al., 2008), hydrothermal (Pei et al., 2009), bio-method (Bai et al., 2009), nano casting method (Xia and Mokaya, 2005), sol-gel (Pinna et al., 2004), combustion (Lima et al., 2006), laser-induced deposition (Zhong et al., 2001), precipitation-gelation (Kim et al., 2004), mechanochemical reaction and subsequent heat treatment (Tsuzuki and McCormick, 2000), chromium oxidation in oxygen (Mougin et al., 2001), and sonochemical methods (Balachandran et al., 1995) have been successfully developed to synthesize Cr_2_O_3_ nanomaterials. However, most of these complex techniques involve specialized lab equipment, high temperatures and are environmentally sensitive (Singh et al., 2019a). Among these methods, co-precipitation is low-cost, convenient, time-saving, and ecologically beneficial synthesis method (Yazid and Joon, 2019; Asha et al., 2021).

This study aims to synthesize pure and Sr (2, 4, and 6 wt%) doped Cr_2_O_3_ nanostructures (NSs) using co-precipitation and analyze their optical, morphological, and structural features, as well as their ability to function as catalyst and antibacterial agent. The catalytic activity of as-prepared NSs was tested against RhB dye reduction, and the bactericidal potential of NSs was examined for MDR *E. coli* (a G-ve bacteria).

## 2 Experimental part

### 2.1 Materials

Chromium acetate (Cr(CH_3_COO)_3_, 99.0%) was purchased from Uni-Chem Chemical Reagents, and strontium chloride hexahydrate (SrCl_2_.6H_2_O, puriss≥99%), sodium hydroxide (NaOH) were purchased from Sigma-Aldrich and used without further purification**.**

### 2.2 Synthesis of Sr-doped chromium oxide

**FIGURE 1:**
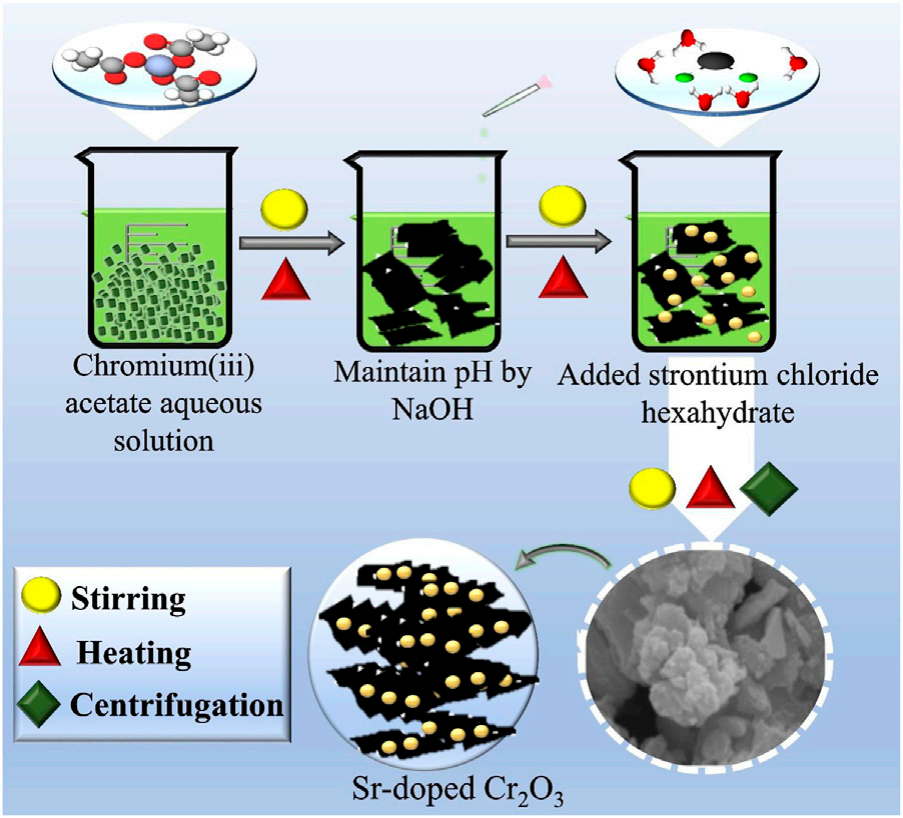
Schematic illustration for synthesis route of pristine and Sr-doped Cr_2_O_3_.

The co-precipitation method was used to synthesize the Cr_2_O_3_ NSs, with 0.5 M of Cr(CH_3_COO)_3_ serving as the precursor material. The pH of the solution was maintained at 12 by adding NaOH drop by drop while constantly stirring at 80 °C. After centrifugation at 7,500 rpm for 6 min to remove impurities, the resulting product was dried at 200°C for 12 h, and a fine powder was obtained using a mortar and pestle. To synthesize Sr-doped Cr_2_O_3_, the same procedure was followed, adding various concentrations of Sr (2, 4, and 6%) in Cr_2_O_3_. The preparation method is illustrated schematically in Figure 1.

### 2.3 Catalytic activity

To assess the catalytic performance of undoped and Sr-doped Cr_2_O_3_ in the presence of the reducing agent sodium borohydride (NaBH_4_), the synthesized NSs were utilized for the reduction of RhB dye, which acts as an oxidizing agent. All the reagents, including RhB and NaBH_4_, were used immediately after preparation to maintain the experimental integrity. Initially, a freshly made 400 µL of 0.1 M NaBH_4_ solution was combined with a 1.5 mL aqueous RhB solution. Subsequently, 400 µL of synthetic nanocatalyst was added and thoroughly mixed with the solution. RhB has a peak absorption at 555 nm, which was chosen to measure the reduction in UV-vis absorption. Adding NaBH_4_ led to a change in RhB to its leuco form, indicating dye reduction. The % reduction was calculated as follows:

$$\%\;reduction=\left(\frac{C_{0}-C_{t}}{C_{0}}\right)\times 100$$

where C_o_ and C_t_ are the initial and specific time absorptions.

### 2.4 Isolation and identification of MDR *Escherichia coli*

#### 2.4.1 Isolation of *Escherichia coli*

To collect unpasteurized milk samples from lactating dairy cows of different farmlands and veterinary clinics in Punjab, Pakistan, prompt milking in a sterile glass container was used. The milk specimen’s transportation proceeded at the temperature of 4°C. The coliform pathogen found in unpasteurized milk was quantified using MacConkey agar. Each plate endured 48 h of incubation at 37°C.

#### 2.4.2 Identification and characterization of bacterial isolates

The variety of Gram stain colonial morphology and biochemical tests were used in conjunction with Bergey’s Manual of Determinative Bacteriology (Holt et al., 1994) to make a preliminary determination of the identity of *E. coli*.

The disc diffusion approach was employed on Mueller Hinton agar (MHA) to investigate antibiotic susceptibility (Bauer, 1966). The test was made to evaluate the antibiotic resistance of gram-ve *E. coli bacteria* against the following antibiotics (classes); Ceftriaxone (Cro) 30 µg (Cephalosporins), Gentamicin (Gm) 10 µg (Aminoglycosides), Ciprofloxacin (Cip) 5 µg (Quinolones), Tetracycline (Te) 30 µg (Tetracyclines), Imipenem (Imi) 10 µg (Carbapenem), Amoxycillin (A) 30 μg (Penicillins), and Azithromycin (Azm) 15 µg (Macrolides). The *E. coli* resistant to 5 μg of the antibiotic ciprofloxacin was conducted through various experiments (Adzitey et al., 2022). Purified cultures of *E. coli* were grown to a turbidity level of 0.5, as determined by the MacFarland standard. Following this, the bacteria were spread out on MHA (Oxoid Limited, Basingstoke, United Kingdom), and antibiotic discs were placed at a distance on the inoculation plate surface. This avoided disrupting inhibition zones. The plates were incubated at 37°C for 48 h while being cultivated, and the data were then examined in accordance with the guidelines provided by the Diagnostic, Therapeutic, and Laboratory Standard Institute (Wayne, 2008). At least three drugs were shown to be ineffective against MDR bacteria (Iwalokun et al., 2004).

### 2.5 Molecular docking analysis

To comprehend the mechanism behind bactericidal action, molecular docking research was conducted on synthetic Cr_2_O_3_ and Sr-doped Cr_2_O_3_ NPs. This was accomplished by focusing on proteins essential for bacterial survival and proliferation. The molecular docking investigation selected several protein targets from biosynthetic pathways, such as dihydrofolate reductase and beta-ketoacyl-acyl carrier protein synthase III (FabH). The dihydrofolate reductase plays a crucial part in synthesizing folic acid, which is essential for the survival of bacteria. FabH enzymes catalyze critical stages in bacterial cells’ fatty acid biosynthesis pathway (Li et al., 2009; Altaf et al., 2020). *E. coli* target protein crystal structures of the high resolution were acquired from the Protein Data Bank. The protein DHFR identified by PDB ID 2ANQ; Resolution: 2.6 (Summerfield et al., 2006) FaBH_*E. Coli*_ with PDB ID 5BNR; Resolution: 1.9 (McKinney et al., 2016) was chosen to comprehend molecular interactions between NPs and protein active pocket residues.

Sybyl-X2.0 was used for molecular docking investigations (Mehmood et al., 2022; Shahzadi et al., 2022). Water molecules and co-crystallized ligands were eliminated to create a protein structure. The protein structures were optimized for energy reduction using default settings and a force field. Following this, a protomol was generated to characterize the binding pocket, and the 10 best-docked conformations were created to investigate the interaction pattern between NPs and active site residues (Ikram et al., 2023).

### 2.6 Characterizations

To analyze the crystal structure and phase information of the NSs, a PANalytical XPert PRO X-ray diffraction (XRD) system was utilized with Cu Kα radiation (λ ∼ 0.0154 nm) within the 2θ range of 20°–70°. The optical properties within the 200–700 nm range were examined using a UV-Vis spectrophotometer (Genesys 10S). The morphology and microstructure of the samples were observed with a JSM-6460LV FE-SEM system with an EDX spectrometer. The PL spectra were collected using a JASCO FP-8300 system. Inter-planar d-spacing of the NSs was measured using the HR-TEM equipment JEOL JEM 2100F.

## 3 Results and discussion

**FIGURE 2:**
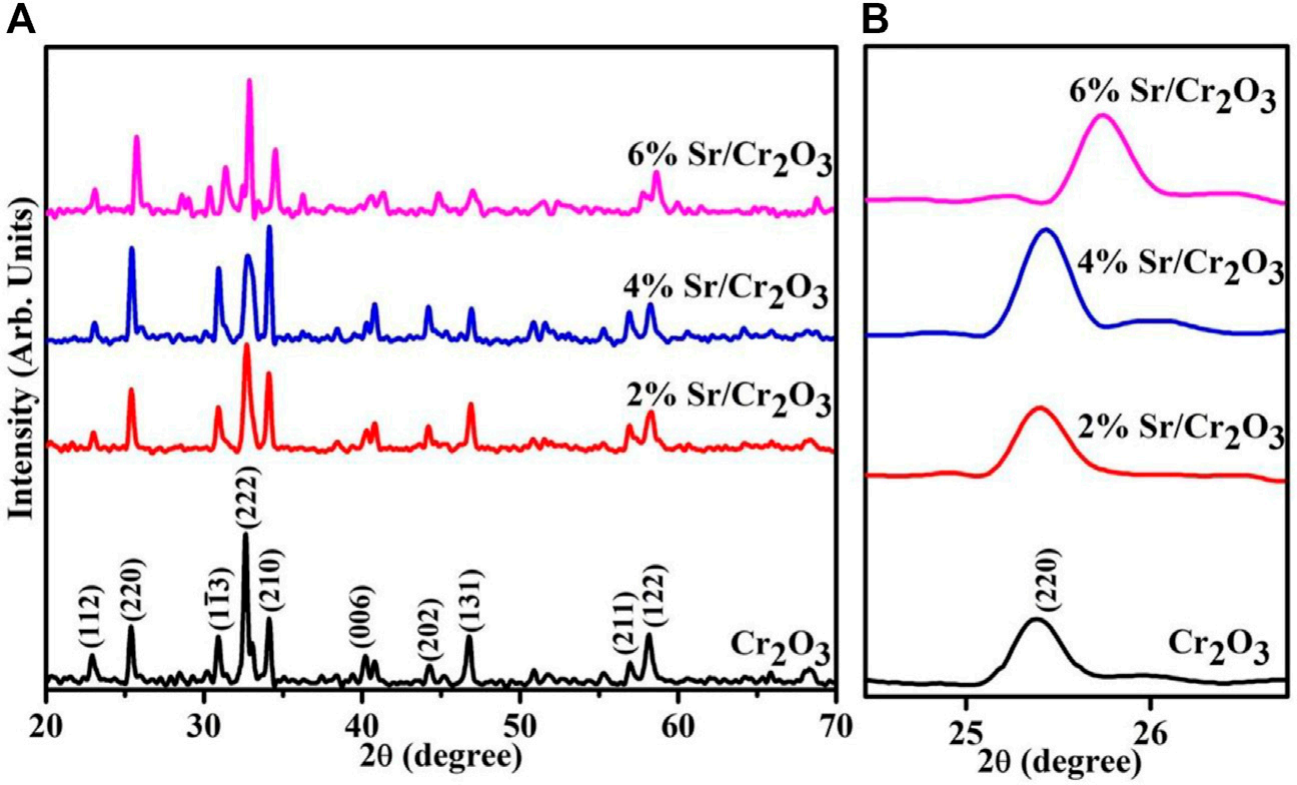
**(A)** XRD patterns of Cr_2_O_3_ and Sr-doped Cr_2_O_3_ **(B)** zoomed area of (220) plane.

The XRD analysis pattern for the phase purity, crystallographic plane structure, and crystallite size of the synthesized products are shown in Figure 2A. The spectra show well-defined peaks at 23.0°, 25.6°, 31.1°, 32.3°, 34.1°, 39.7°, 44.1, 46.9°, 57.1°, and 58.3°, which correspond to the (112), (220), (1 $\bar{1}$ 3), (222), (210), (006), (202), (131), (211), and (122) facets that are well matched with standard spectrums (00-038-1479/00-036-1329/80-2473). These planes belong to polycrystalline Cr_2_O_3_, Cr_2_O_5,_ and Cr_8_O_13_, which have been previously reported (Ivanov et al., 2001; Panda et al., 2018). Norby et al. examined the crystal structure of Cr_8_O_13_ in detail (Norby et al., 1991). Cr_8_O_21_ is composed of two CrO_6_ octahedra that share a standard edge. Two chromate groups (CrO_4_, tetrahedra) connected the double octahedra to form a sheet. Finally, tetrachromate groups (Cr_4_O_13_) link these sheets to construct a three-dimensional structure. Upon Sr doping, the slight shift of peaks is caused by extensive dispersion of the dopant element between the interlayers of the host sample. The size of the crystallite affects the crystallinity-dependent properties of the crystal. Moreover, larger crystallites produce sharper peaks in the XRD pattern for a particular crystal plane. The shift in a peak that occurs due to doping is attributed to the presence of Sr in the host of the Cr_2_O_3_ matrix (see Figure 2B). Crystallite size is correlated with the width of a peak. Using the following Debby-Scherrer formula (Abdullah et al., 2014), the crystallite size (D) of pristine Cr_2_O_3_ and (2, 4, and 6%) Sr-doped Cr_2_O_3_ was found to be 37.5 nm, 28.1 nm, 33.9 nm, and 56.6 nm respectively.

**FIGURE 3:**
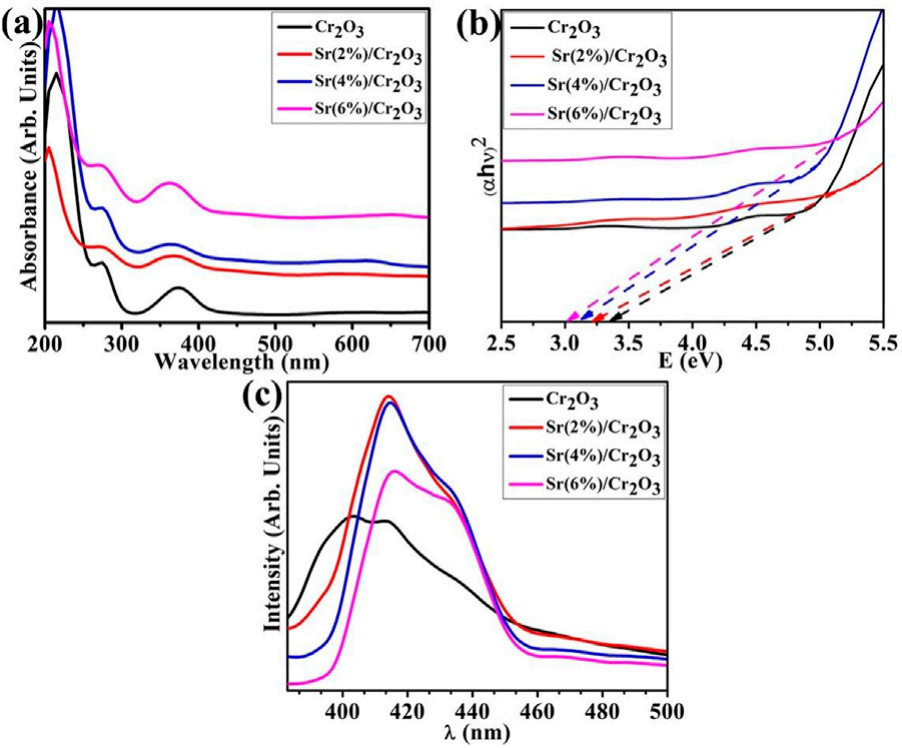
**(A)** optical spectra, **(B)** band gap energy determination, and **(C)** PL emission spectra of Cr_2_O_3_ and Sr-doped Cr_2_O_3_.

The absorption spectra of the synthesized Cr_2_O_3_ and Sr-doped Cr_2_O_3_ nanomaterials from the UV-Vis spectrophotometer are presented in Figure 3A. On the graph, two significant absorption peaks were demonstrated at 260 nm and 360 nm wavelengths. The peak showed a Cr_2_O_3_ NSs band gap transition at 360 nm (Singh et al., 2019b). In optical characteristics, the estimate of band gap energy is an essential factor. There are numerous ways to calculate the optical band gap. Among them, the optical procedure is the most precise and simple way to detect the band gap energy of materials (Ashiri et al., 2009). The Tauc equation interprets the relationship between the absorption coefficient (α) and the incident energy (hν), which was used to obtain the optical band gap energy of the materials. The optical band gap was determined using the Tauc relation:

$${\left(\alpha h\nu \right)}^{2}=B\left(h\nu -Eg\right)$$

where hν is the photon energy, E.g., represents the optical band gap, and B is constant and takes on different values depending on the transitions. Therefore, the band gap can be determined by extrapolating the linear portion of the curve intersecting the hν axis. The Cr_2_O_3_ NSs prepared by co-precipitation contain a direct band gap (Singh et al., 2019b). As it is obvious from Figure 3B, doping substantially redshifted the band gap energy from 3.3 eV in pristine Cr_2_O_3_ to around 3.0 eV in the case of (2,4% and 6%) Sr-doped Cr_2_O_3_ attributed to quantum confinement effect. The incorporation of dopants into Cr_2_O_3_ has modified the optical band gap and crystallinity, indicating the interaction and complexation of dopants with the Cr_2_O_3_.

The Cr_2_O_3_ NSs photoluminescence (PL) spectra are displayed in Figure 3C. The PL spectra were used to analyze the charge recombination of the NSs. The broad emission peaks could be observed in all samples, attributed to higher crystallinity of the as-synthesized NSs. It is shown that the Sr-doped Cr_2_O_3_ powders have a greater PL signal than pure Cr_2_O_3_ (Figure 5), demonstrating increased charge recombination. The most prevalent defect sites in oxide materials are oxygen vacancies, that produce PL emission by recombining with an electron. The Sr doping causes increased PL intensities compared to Cr_2_O_3_ (Kamari et al., 2019). The transition involving the 3d^3^ electrons of the Cr^3+^ ions causes the peak to appear at ∼ 425 nm. The oxygen interstitials, oxygen vacancies, chromium interstitials, dangling surface bonds, and chromium vacancies may all be responsible for the broad emission peaks in the visible spectrum range that are centered at roughly 415 nm (violet PL) and 435 nm (blue emission) (Almontasser and Parveen, 2020).

**FIGURE 4:**
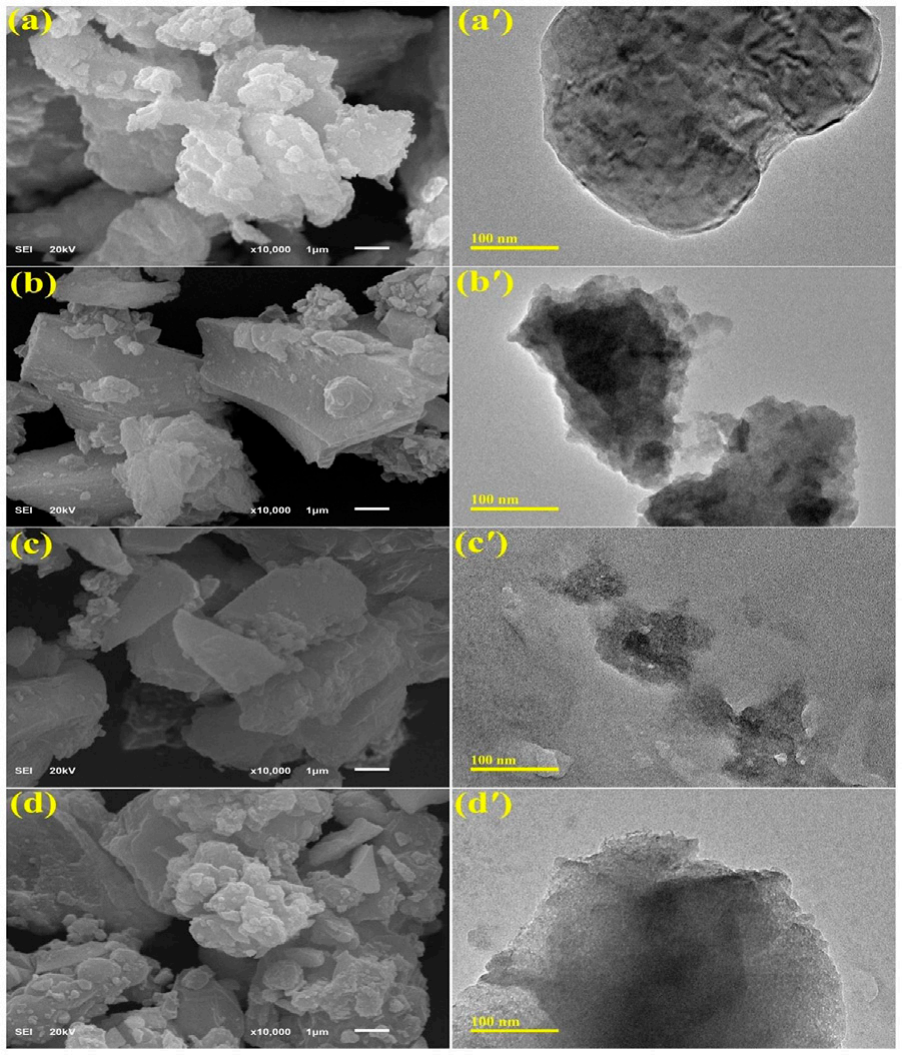
FE-SEM and TEM images of **(A, A′)** Cr_2_O_3_, **(B, B′)** 2% Sr/Cr_2_O_3_, **(C, C′)** 4% Sr/Cr_2_O_3_, **(D, D′)** 6% Sr/Cr_2_O_3_.

The morphology of as-grown material was assessed using the FE-SEM and TEM techniques. Figure 4 illustrates the typical morphology of Cr_2_O_3_ and Sr (2%, 4%, and 6%) doped Cr_2_O_3_. The FE-SEM and TEM micrographs demonstrate that most of Cr_2_O_3_ consist of strongly agglomerated NPs with a morphology resembling nanoflakes, as depicted in Figure 4(A-A′). Nanoflakes are preferable for various applications demanding higher redox-active sites since they tend to give more surface area for interfacial contact (Rashad et al., 2020). Sr doping caused NPs to agglomerate further, as shown in Figure 4(B-B′, D-D′). Consequently, the presence of Sr in the lattice of Cr_2_O_3_ increased the roughness of resulting NSs. As reported earlier, the agglomeration of NPs is caused by high surface area and high surface energy (Anbu et al., 2022). This rise in NSs surface area, caused by Sr doping, promotes the formation of reactive oxygen species (ROS), thereby improving the antibacterial activity (Yarahmadi et al., 2021).

**FIGURE 5:**
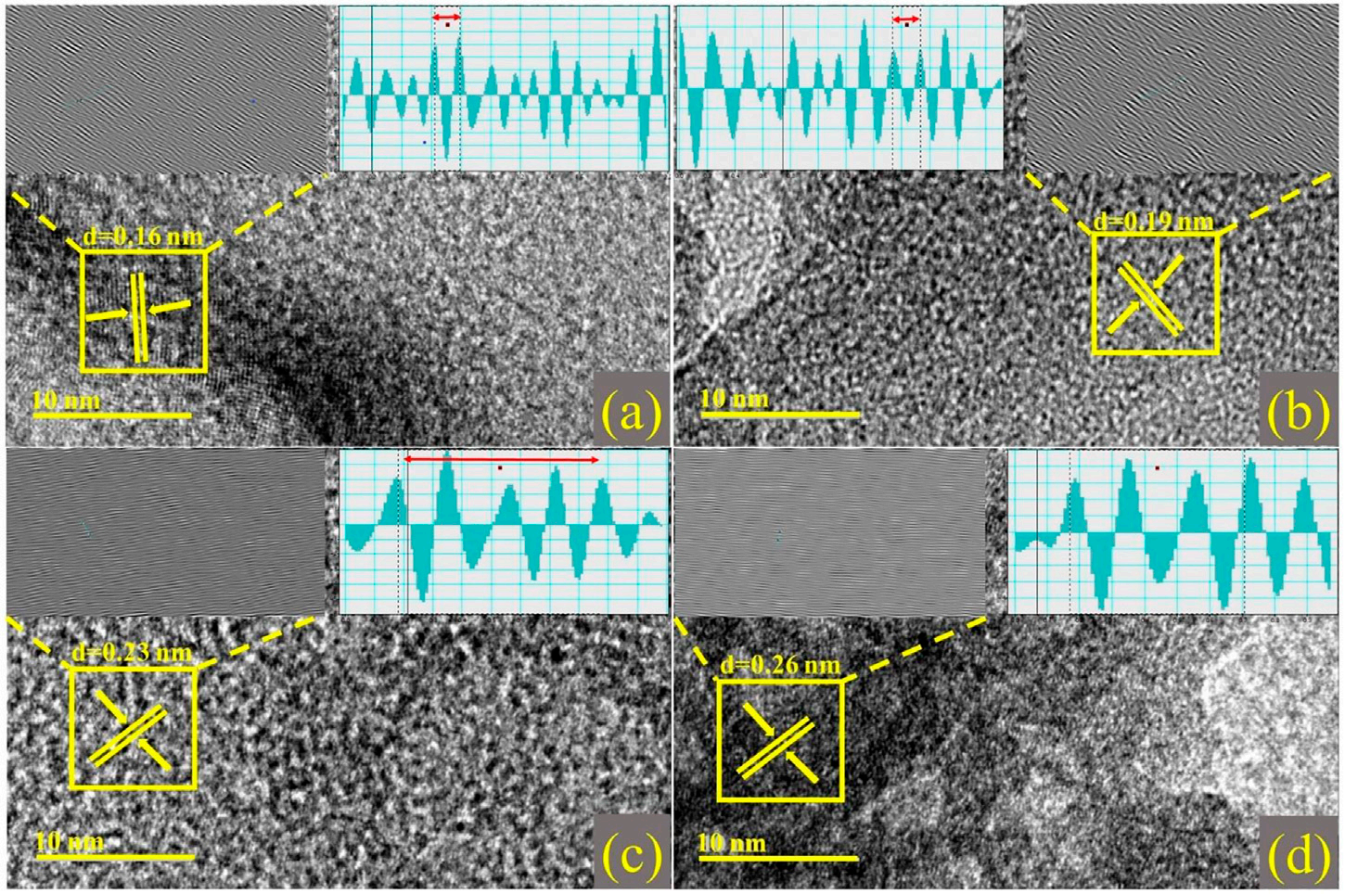
d-spacing calculated from HR-TEM images with Inverse Fast Fourier Transform (IFFT) and IFFT image profile of **(A)** Cr_2_O_3_ **(B)** 2% Sr/Cr_2_O_3_ **(C)** 4% Sr/Cr_2_O_3_ **(D)** 6% Sr/Cr_2_O_3_.

The number of layers can be microscopically estimated using a high-resolution TEM (HR-TEM) study of edge regions. HR-TEM pictures show many atomic planes exhibiting periodic atomic configurations on a single grain, as illustrated in Figures 5A–D. Moreover, planes are well arranged to form a single layer at particular points, with an interplanar spacing of 0.16 nm. This correlates with the XRD-determined (2 1 1) facet of the rhombohedral Cr_2_O_3_ phase. The addition of dopants resulted in samples with d-spacings of 0.19, 0.23, and 0.26 nm, as indicated by XRD analysis.

**FIGURE 6:**
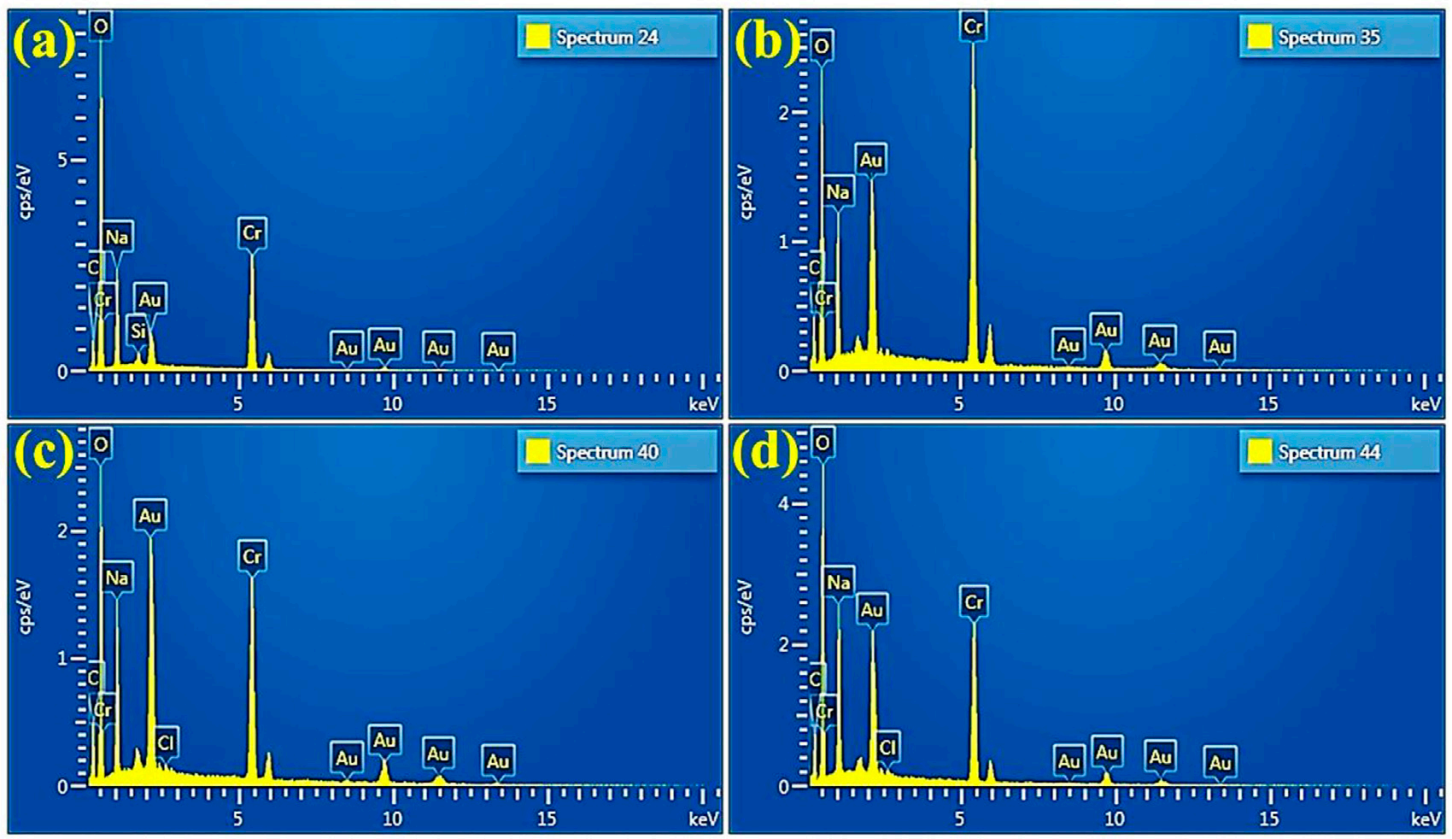
EDS of **(A)** Cr_2_O_3_ **(B)** 2% Sr/Cr_2_O_3_ **(C)** 4% Sr/Cr_2_O_3_ **(D)** 6% Sr/Cr_2_O_3_.

Energy dispersive spectroscopy (EDS) was used to investigate the elemental composition of as-prepared Cr_2_O_3_ NSs (Figure 6). The spectrum demonstrates the corresponding peaks for chromium and oxygen, along with minor Au peaks. Additional Na peaks were also noticed, which could have resulted from using NaOH during the synthesis process. Furthermore, no Sr peaks were observed in EDS attributed to low concentration of dopants.

**FIGURE 7:**
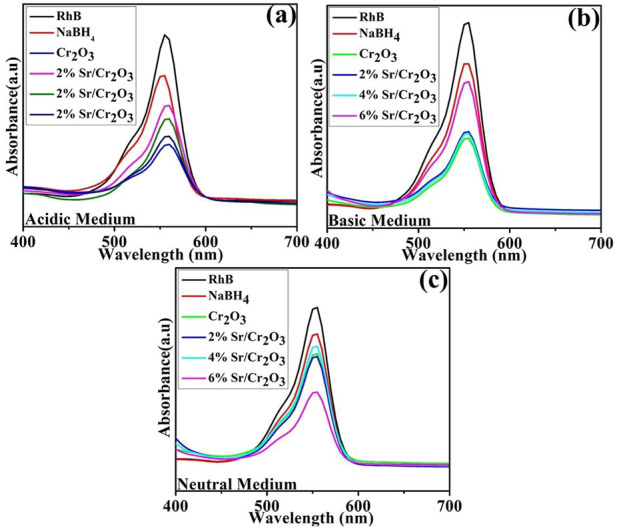
UV-Visible absorption spectra of RhB in the presence of nanocatalysts. **(A)** Acidic Medium, **(B)** Basic Medium, **(C)** Neutral Medium.

The catalytic activity of nanocatalysts against RhB dye was investigated utilizing NaBH_4_ as a reducing agent. The visible absorption spectra of RhB solution during the reduction process are displayed in Figures 7A–C. The absorption peak at 555 nm is reduced after the nanocatalyst is added, indicating that the dye molecules have undergone reduction.

**FIGURE 8:**
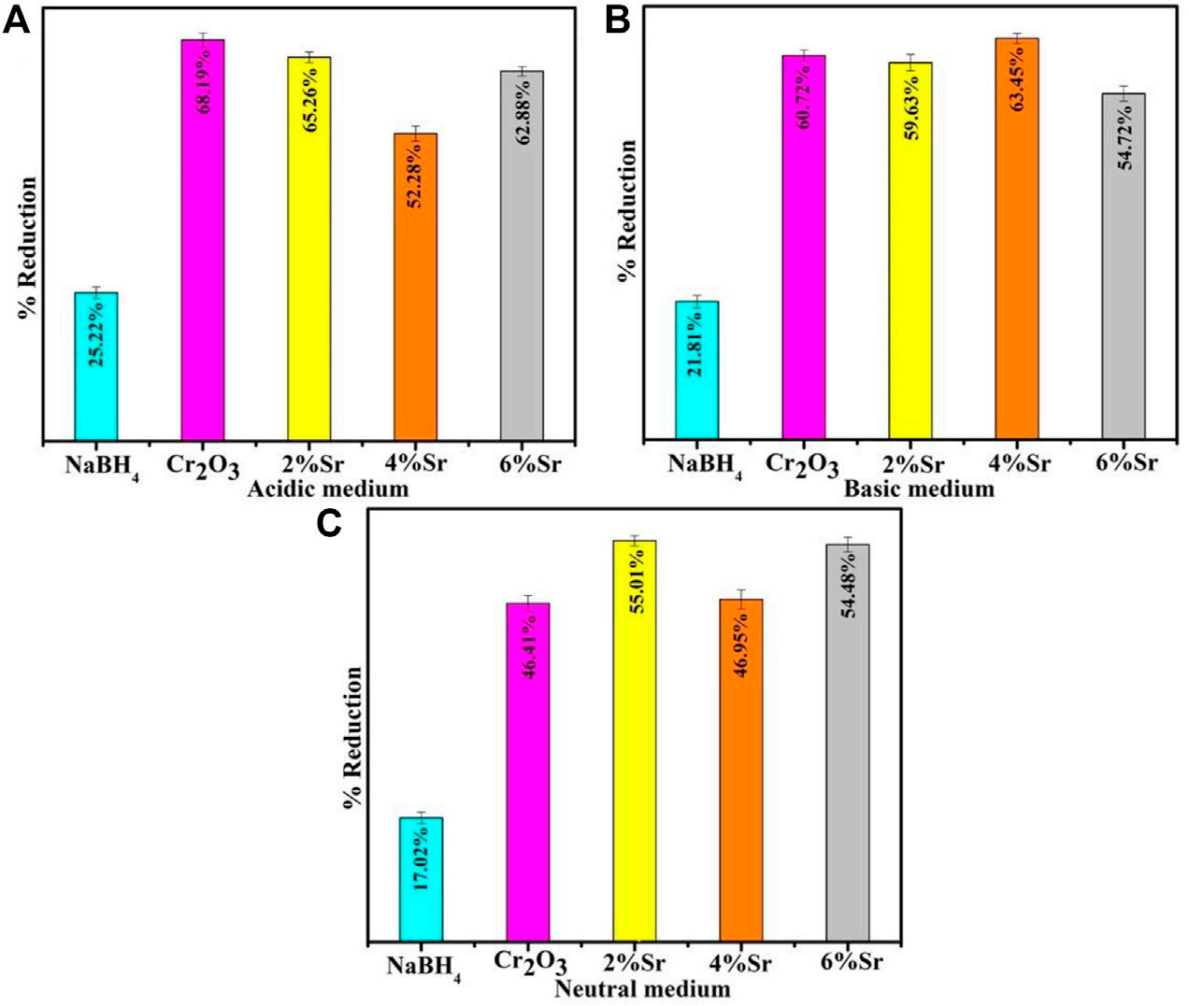
Catalytic activity of Cr_2_O_3_ and Sr-doped Cr_2_O_3_ in the presence of NaBH_4_. **(A)** Acidic Medium, **(B)** Basic Medium, **(C)** Neutral Medium.

UV–vis absorption spectra of the RhB solution treated in the three different pH (acidic, basic, and neutral) show that dye reduction was significant at pH = 4, well matched with previous observations (Cui et al., 2015). The pristine Cr_2_O_3_ and (2,4% and 6%) Sr-doped Cr_2_O_3_ NSs showed maximum % reduction of 68.19%, 65.26%, 52.28%, and 62.88% in acidic medium (pH = 4), 60.72% 59.63%, 63.45%, 54.72% in basic medium (pH = 12) and 46.41%, 55.01%, 46.95% and 54.48% in neutral medium (pH = 7) respectively as shown in Figures 8A–C. In the absence of a catalyst, the reduction process occurred at a slow rate, resulting in only 25.22%, 21.81%, and 17.02% reduction in acidic, basic, and neutral environments, respectively. The effects of Sr as dopant reduced the effects of Cr_2_O_3_ as nanocatalyst in acidic and basic mediums. At pH = 8, 6% Sr-doped Cr_2_O_3_ causes an increase in catalytic activity from 46.41% to 54.48%. The shape, size, and surface area of nanocatalysts significantly impact performance reduction by generating substantial active sites. The undoped catalyst showed a better reduction rate than the Sr-doped nanomaterial. Due to their various placements within the host lattice, dopants may not have identical impacts on trapping electrons on the interface or during interfacial charge transfer (Munusamy et al., 2013). Moreover, dopants take up residence in the host material active sites, reducing the adsorption process—consequently, the catalytic efficiency change with the choice of dopant and morphology. The decolorization mechanism of RhB by Cr_2_O_3_ depends on the crystal structure of the oxides and the solution pH.

**FIGURE 9:**
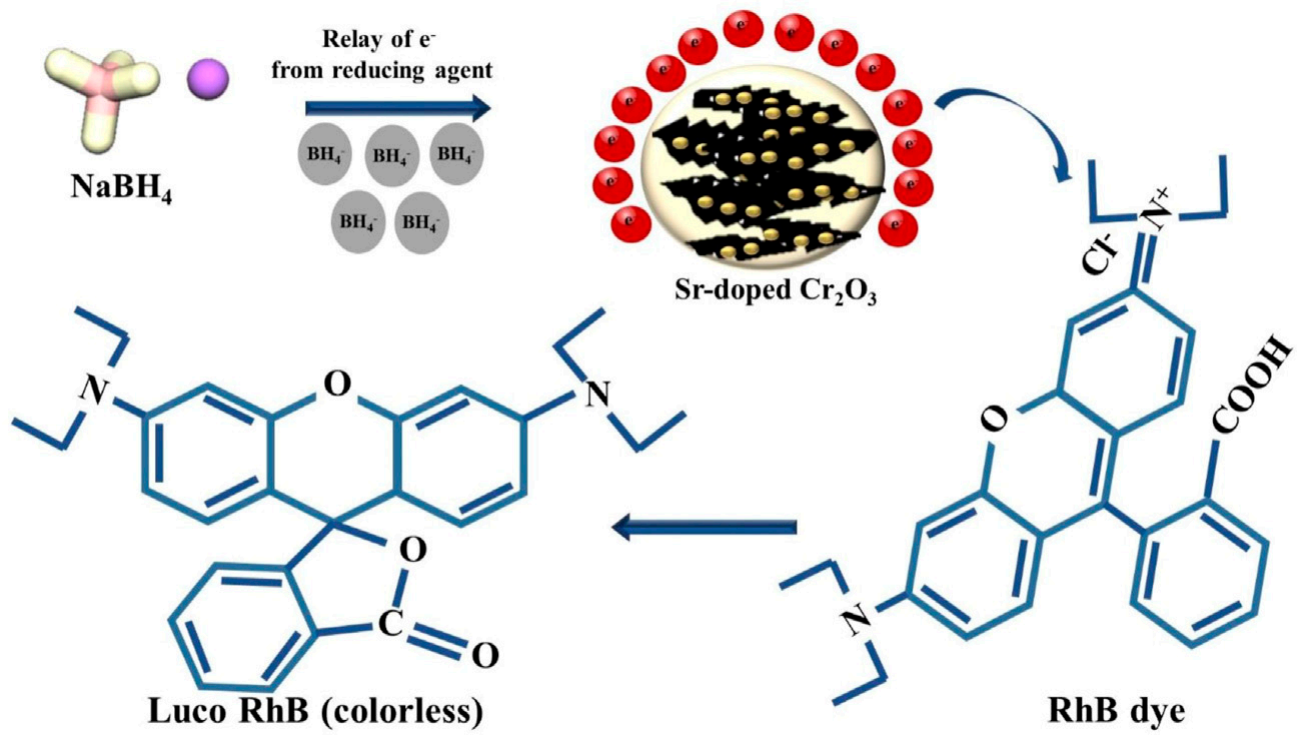
Schematic illustration of catalytic activity.

The electrochemical mechanism can explain the catalytic process of the reduction reaction using NSs. The reduction process begins with the migration of BH^4−^ from NaBH_4_ and RhB molecules via aquatic solution to the exterior of Sr-doped Cr_2_O_3_. Afterward, the nanocatalysts on the top of the heterogeneous catalyst act as an electron relay system to accelerate the flow of electrons from the donor to the acceptor, i.e., from BH^4−^ to RhB. The next step entails a nanocatalyst’s catalytic decomposition of the hydrogen source NaBH_4_ to deliver hydrogen atoms. The produced reactive hydrogens subsequently react with dye molecules, causing the breakdown of RhB molecules into its luco form (Alani et al., 2021), as shown in Figure 9. The nanocatalysts enhanced the reduction of RhB with NaBH_4_, resulting in significant reduction efficiency.

*In-vitro* antibacterial activity of Cr_2_O_3_ and (2, 4, and 6%) Sr-doped Cr_2_O_3_ was evaluated by assessing inhibitory zones against Gram-negative bacteria MDR *E. coli* with agar-based diffusion technique (see Table 1). Significant inhibitory zones were found at lower and higher doses (8.65—10.15 mm) and (9.65—11.75 mm) against MDR *E. coli,* respectively. Ciprofloxacin showed an 11.85 mm inhibition zone compared to DI water (0 mm). The formation of ROS is enhanced by the Sr-doping, that increases the specific surface area of Cr2O3 NPs, thereby improving the antibacterial activity of the NSs. Additionally, the uptake of positively charged metal ions Cr3+ following their release from Cr2O3 at the cell boundary might lead to bacterial cell death by increasing their localized concentration. The outcomes of the current study are consistent with previous research that revealed metal oxide NPs have superior action against Gram-negative pathogens (Ahmad et al., 2014; Yarahmadi et al., 2021). This is due to the fast passage of smaller-sized NPs through the cell wall of *E. coli* (a Gram-negative bacterium), which has a single peptidoglycan layer, in contrast to the cell wall of Gram-positive bacteria, which has multiple peptidoglycan layers. Consequently, the outer membrane of bacteria enables greater NPs penetration to the bacterial cell wall.

**TABLE 1:** The bactericidal potential of Cr_2_O_3_ and Sr-doped Cr_2_O_3_.

Inhibition zone (mm)		
Samples	0.5 mg/50 µL	1.0 mg/50 µL
Cr_2_O_3_	8.65	9.65
2% Sr/Cr_2_O_3_	9.05	10.45
4% Sr/Cr_2_O_3_	9.55	10.95
6% Sr/Cr_2_O_3_	10.15	11.75
Ciprofloxacin	11.85	11.85
DI water	0	0

Numerous mechanisms have been recognized as being responsible for antibacterial action. In the vicinity of air and metallic nanoclusters (as Cr_2_O_3_ in our case), reactive oxygen species (ROS), including reactive nitrogen species and hydrogen peroxide, are generated. Examples of ROS include free radicals (•OH, ^1^O_2_), small molecules (such as H_2_O_2_), and superoxide ions (such as −O_2_) (Rashad et al., 2020). It has also been observed that physicochemical properties, such as crystal structure, defects, surface charge, and composition, are directly correlated with the improved antimicrobial effect of materials. Specifically, it has been discovered that NPs of smaller size are substantially more effective antibacterial agents. As a result of their disintegration, harmful metal ions can infiltrate bacterial cells, making them a more effective tool against bacteria. NPs with a high metal oxide content, such as Sr-doped Cr_2_O_3_ NSs, may accumulate on the surface of bacteria if they are encased in nanoflakes. During contact, the rough surface of Sr-doped Cr_2_O_3_ encloses the bacterial surface.

**FIGURE 10:**
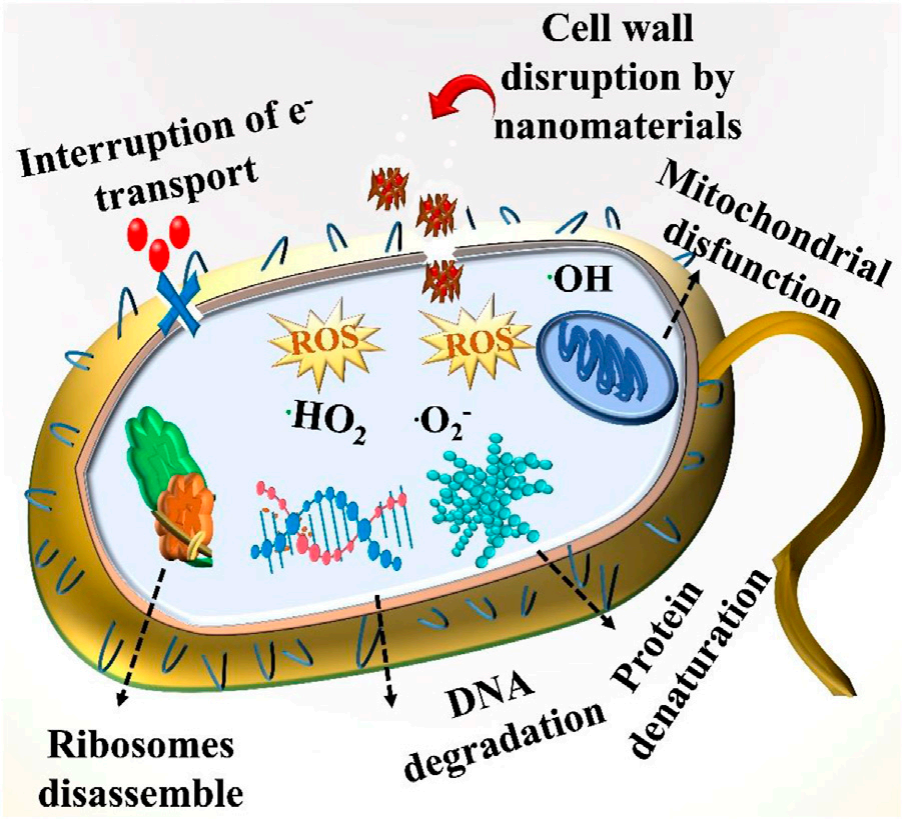
Antibacterial action mechanism of synthesized NSs.

The Sr-doped Cr_2_O_3_ NSs react oxygen molecules with electrons to produce superoxide ions (^**.**^O_2_^−^). The ^**.**^ HO_2_ can be produced by reacting ^**.**^ O_2_^−^ with hydrogen ions. Hydrogen peroxide (H_2_O_2_) can be produced by the interaction of ^**.**^ HO_2_ with hydrogen ions. Following this, ^**.**^HO_2_ and H_2_O_2_ can react to generate extremely reactive hydroxyl radicals (^**.**^OH). The presence of such particles leads to protein dysfunction, DNA damage, cell membrane deterioration, and an increase in death receptor gene expression. The interaction of metal oxide NPs with the thiol groups present in essential enzymes for bacterial survival results in the death of bacterial cells, as shown in Figure 10. Antibacterial action also involves the inhibition of membrane function. The electrostatic interaction of metal NPs with the exterior of the microorganisms also triggers this process. This results in the accumulation of NPs on the cell’s surface and a change in the structure of the cell, both of which inhibit the growth of bacterial cells (Alahmadi et al., 2017; Almontasser et al., 2021).

**FIGURE 11:**
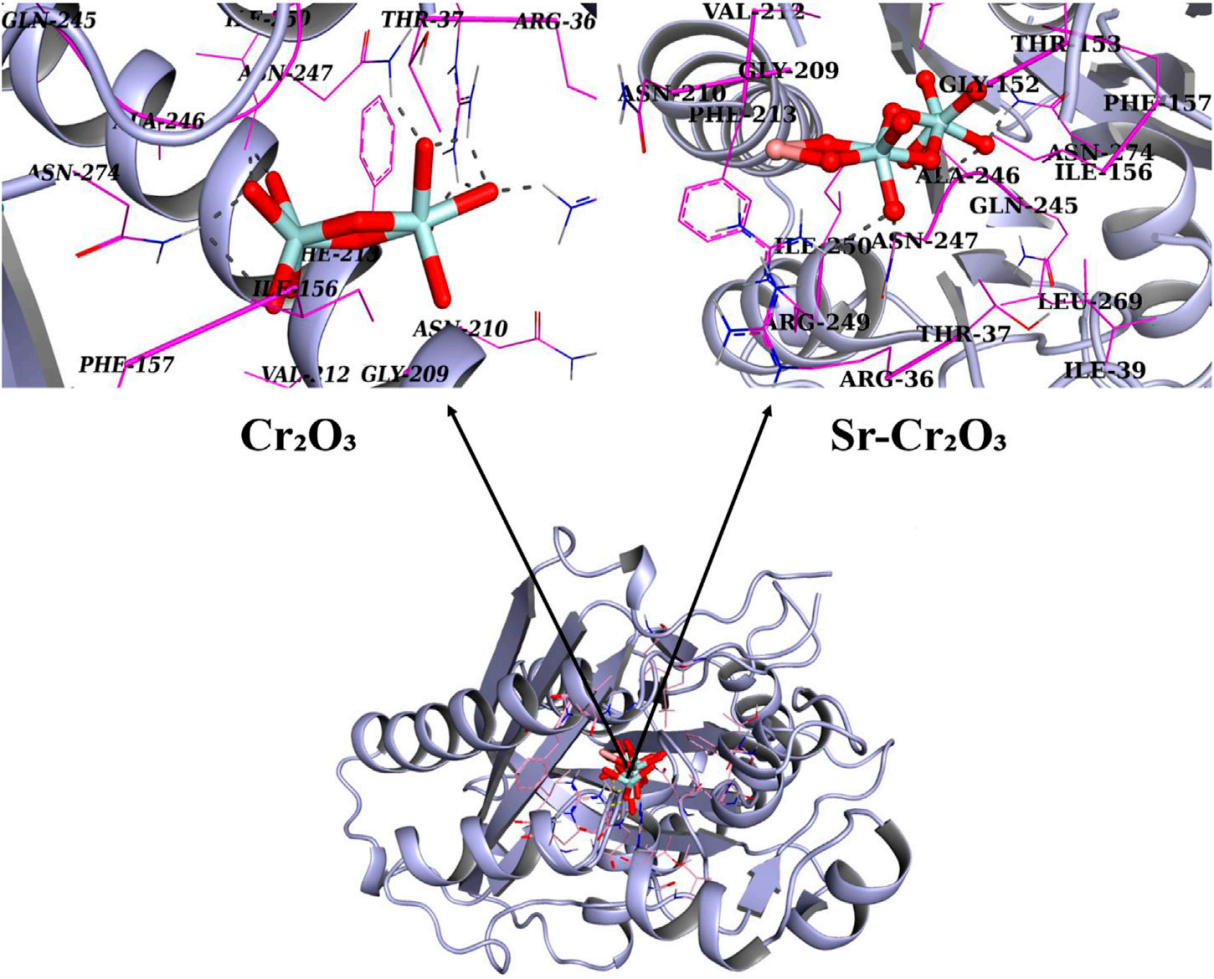
3D graphical representation of binding interaction patterns of Cr_2_O_3_ and Sr-doped Cr_2_O_3_ nanocomposites inside active pockets of FabH from *E. Coli* (FabH_*E. Coli*_).

To understand the possible molecular and atomic-level mechanisms responsible for the antibacterial activity of NPs, it is essential to examine their binding interactions with potential protein targets. The enzyme targets for this investigation relate to metabolic pathways critical for bacterial survival and growth. Molecular docking analysis was conducted to determine the binding interaction pattern of Cr_2_O_3_ and Sr-doped Cr_2_O_3_ with different *E. coli* enzyme targets. β-ketoacyl-acyl carrier protein synthetase III (FabH)_*E. coli*_ formed the best-docked complexes with Cr_2_O_3_ (see Figure 1). The optimal Cr_2_O_3_-FabH_*E. Coli*_ docking arrangement has a docking score of 7.07. Cr_2_O_3_ established H-bonding interactions with Arg36, Thr37, Asn247, and Asn274, as seen in Figure. In addition, Sr-doped Cr_2_O_3_ NPs exhibit H-bonding interaction with Asn247, Arg249, and Asn274, leading to a bind score of 8.62 when docked into the active pocket of FabH, as shown in Figure 11.

**FIGURE 12:**
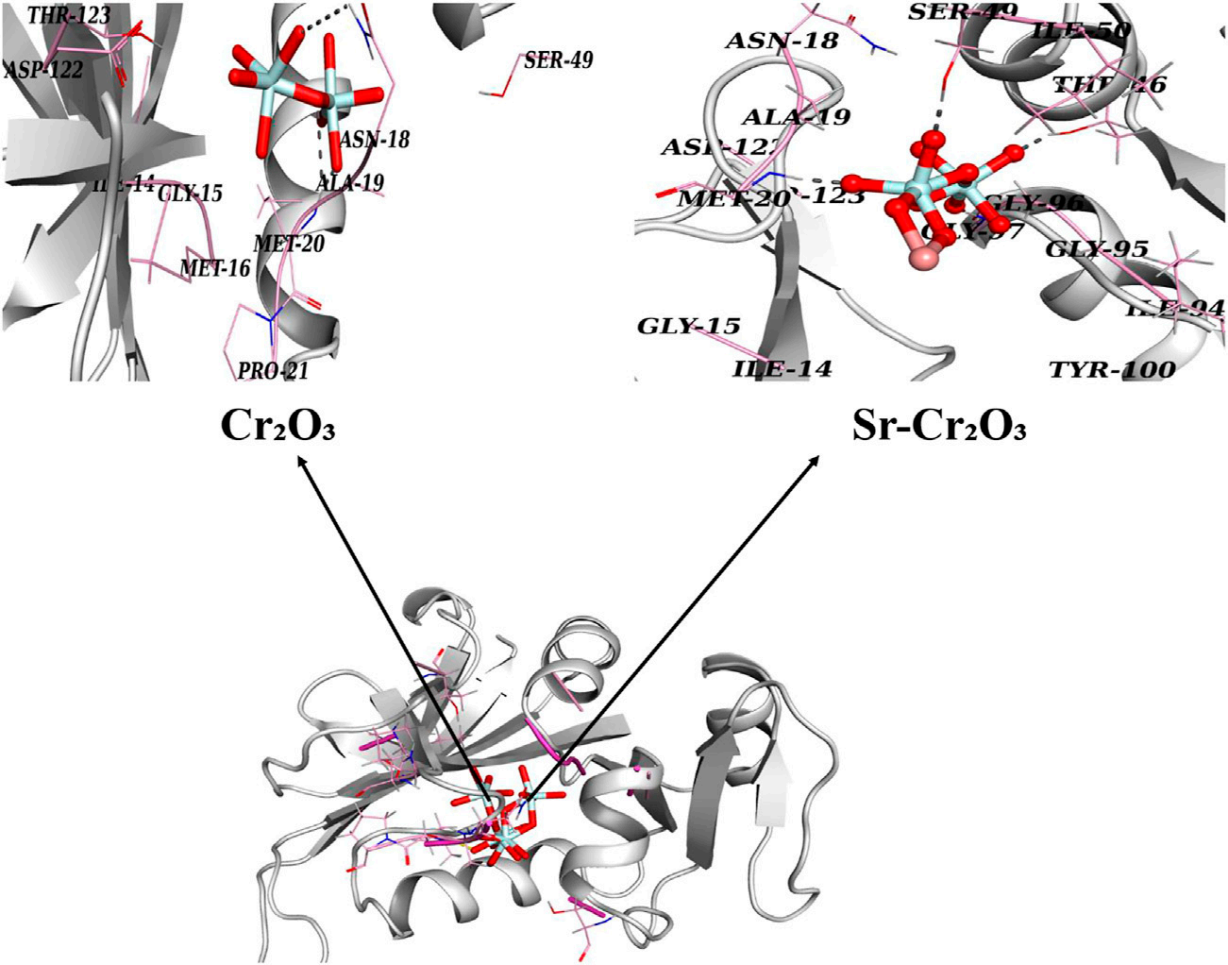
3D graphical representation of binding interaction patterns of Cr_2_O_3_ and Sr-doped Cr_2_O_3_ nanocomposites inside active pockets of DHFR from *E. Coli* (DHFR_*E. Coli*_).

In the case of DHFR_*E. Coli*_, Cr_2_O_3_ exhibited hydrogen-bonding interactions with Asn18 and Met20, with a binding score of 8.78. Similarly, Sr-doped Cr_2_O_3_ nanocomposites also showed comparable binding interactions and scores with active site residues. In the case of Sr-doped Cr_2_O_3_, residues interacting through H-bonds were Met20, Thr46, Ser49, and Gly97, with active pockets having an overall binding score of 9.88, as depicted in Figure 12.

## 4 Conclusion

The co-precipitation technique was used to prepare pristine and Sr-doped Cr_2_O_3_ NSs for catalytic and antibacterial applications. The properties of synthesized NSs were investigated using various structural and optical characterization techniques. The XRD analysis endorsed the polycrystalline planes of pristine Cr_2_O_3_ and crystalline size increases from 37.5 nm to 56.6 nm in the case of 6% Sr-doped Cr_2_O_3_. FE-SEM and TEM micrographs indicate that the presence of Sr in the lattice of Cr_2_O_3_ increased the roughness of resulting NSs attributed to high surface area and high surface energy. Sr doping enhances the surface area of NPs, leading to a more significant formation of reactive oxygen species and, ultimately, a higher antibacterial effect. The interlayer spacing (0.16–0.26 nm) in pure and Sr-doped materials was consistent with HR-TEM. The optical spectra of the samples indicate a redshift after doping, causing a reduction in, E_.g.,_ from 3.3 to 3.0 eV, as revealed by UV–vis spectroscopy. Regarding the reduction efficiency against RhB, the synthesized nanocatalysts demonstrated that the pristine Cr_2_O_3_ exhibited superior catalytic activity compared to the Sr-doped nanomaterials. *In-vitro* antibacterial activity of NSs using an agar-based diffusion technique shows that significant inhibition zone measurements were 8.65—10.15 mm and 9.65—11.75 mm for lower and higher concentrations against MDR *E. coli,* respectively. Cr_2_O_3_ and Sr-doped Cr_2_O_3_ were shown to have an impressive binding score and interaction mechanism within the active region of targeted proteins, indicating that they may be employed as a possible inhibitor of FabH and DHFR enzymes and warranting further exploration into their inhibitory properties. The superior inhibitory activity observed against MDR bacteria classifies these samples as viable candidates for wastewater treatment systems.

## Data Availability

The original contributions presented in the study are included in the article/supplementary material, further inquiries can be directed to the corresponding authors.
