# Corrigendum: Strontium-doped chromium oxide for RhB reduction and antibacterial activity with evidence of molecular docking analysis

**DOI:** 10.3389/fchem.2023.1228244

**Published:** 2023-05-31

**Authors:** Muhammad Ikram, Anum Shahzadi, Muhammad Bilal, Ali Haider, Anwar Ul-Hamid, Walid Nabgan, Junaid Haider, Salamat Ali, Francisco Medina, Muhammad Imran

**Affiliations:** ^1^ Solar Cell Applications Research Lab, Department of Physics, Government College University Lahore, Lahore, Pakistan; ^2^ Faculty of Pharmacy, The University of Lahore, Lahore, Pakistan; ^3^ Department of Clinical Sciences, Faculty of Veterinary and Animal Sciences, Muhammad Nawaz Shareef University of Agriculture, Multan, Pakistan; ^4^ Core Research Facilities, King Fahd University of Petroleum and Minerals, Dhahran, Saudi Arabia; ^5^ Departament d’Enginyeria Química, Universitat Rovira i Virgili, Tarragona, Spain; ^6^ Chinese Academy of Sciences, Tianjin Institute of Industrial Biotechnology, Tianjin, China; ^7^ Department of Physics, The University of Lahore, Lahore, Pakistan; ^8^ Government College University Faisalabad, Sahiwal, Punjab, Pakistan

**Keywords:** dye degradation, MDR *E. coli*, RhB, antibacterial, Cr_2_O_3_

In the published article, there was an error in the author list. Author Francisco Medina was erroneously excluded. The corrected author list appears above.

The **Author Contributions** statement has also been updated to reflect the addition of this author and is now as follows:

“MIk: Conceptualization, Investigation, Writing—original draft preparation, Supervision, Funding acquisition, Visualization. AS: Methodology, Formal analysis, Writing—review and editing, MB: Conceptualization, Resources. AH: Investigation, Data Curation. AU-H: Methodology, Investigation. WN: Conceptualization, Writing—review and editing, Investigation. JH: Formal analysis, Resources. SA: Conceptualization, Data Curation. FM: data analysis, interpretation, conceptualization, review and editing, investigation, formal analysis, and fund acquisition. MIm: Methodology, Formal analysis”.

In the published article, there was an error in the **Acknowledgments** statement. Some of the funding details were missed. The correct statement appears below:

“The authors express their gratitude to the Higher Education Commission (HEC), Pakistan, for the support through NRPU 20-17615 (MIk) and to the Universitat Rovira i Virgili for the support under the Maria Zambrano Programme (Reference number: 2021URV-MZ-10). Authors are also thankful to Proyectos de Generación de Conocimiento AEI/MCIN (PID2021-123665OB-I00) the project reference number of TED2021-129343B-I00 Grant PID2021-123665OB-I00 and TED2021-129343B-I00 funded by MCIN/AEI/ 10.13039/501100011033 and, as appropriate, by “ERDF A way of making Europe”, by the “European Union” or by the “European Union NextGenerationEU/PRTR”.

The authors apologize for these errors and state that this does not change the scientific conclusions of the article in any way. The original article has been updated.

